# Intraocular Pressure and Associations in Children. The Gobi Desert Children Eye Study

**DOI:** 10.1371/journal.pone.0109355

**Published:** 2014-10-08

**Authors:** Da Yong Yang, Kai Guo, Yan Wang, Yuan Yuan Guo, Xian Rong Yang, Xin Xia Jing, Hai Ke Guo, Yong Tao, Dan Zhu, Jost B. Jonas

**Affiliations:** 1 Southern Medical University, Guangzhou, Guangdong, China; 2 Department of Ophthalmology, Guangdong General Hospital/Guangdong Academy of Medical Sciences, Guangzhou, Guangdong, China; 3 The Affiliated Hospital of Inner Mongolia Medical University, Hohhot, Inner Mongolia, China; 4 Department of Ophthalmology, People's Hospital, Peking University, & Key Laboratory of Vision Loss and Restoration, Ministry of Education, Beijing, China; 5 Department of Ophthalmology, Medical Faculty Mannheim of the Ruprecht-Karls-University Heidelberg, Mannheim, Germany; University of Missouri-Columbia, United States of America

## Abstract

**Purpose:**

To assess the intraocular pressure (IOP) and its association in children in a population living in an oasis in the Gobi Desert.

**Methods:**

The cross-sectional school-based study included all schools in the Ejina region. The children underwent an ophthalmic examination, non-contact tonometry and measurement of blood pressure and body height and weight.

**Results:**

Out of eligible 1911 children, 1565 (81.9%) children with a mean age of 11.9±3.5 years (range: 6–21 years) participated. Mean spherical refractive error was −1.58±2.00 diopters. In multivariate analysis, higher IOP (right eye) was associated with younger age (*P*<0.001; standardized coefficient beta: −0.13; regression coefficient B: −0.13; 95% Confidence interval (CI):−0.18, −0.07), higher diastolic blood pressure (*P*<0.001;beta:0.13;B:0.05;95%CI:0.03,0.07), higher corneal refractive power (*P*<0.001;beta:0.11;B:0.23;95%CI:0.12,0.34), more myopic refractive error (*P* = 0.035;beta: −0.06;B: −0.10;95%CI: −0.19, −0.001), and Han Chinese ethnicity of the father (*P* = 0.03;beta:0.06;B:0.42;95%CI:0.04,0.89). If age and diastolic blood pressure were dropped, higher IOP was associated with higher estimated cerebrospinal fluid pressure (CSFP) (*P*<0.001;beta:0.09; B:0.13;95%CI:0.06,0.21) after adjusting for higher corneal refractive power (*P*<0.001) and Han Chinese ethnicity of the father (*P* = 0.04). Correspondingly, higher IOP of the left eye was associated with younger age (*P*<0.001;beta: −0.15;B: −0.16;95%CI: −0.21, −0.10), female gender (*P*<0.001;beta:0.09;B:0.65;95%CI:0.30,1.01), higher corneal refractive power (*P*<0.001;beta:0.08;B:0.19;95%CI:0.06,0.32), more myopic refractive error (*P* = 0.03;beta: −0.06;B: −0.12;95%CI: −0.22, −0.01), and higher estimated CSFP (*P*<0.001;beta:0.11;B:0.17;95%CI:0.09,0.24).

**Conclusions:**

In school children, higher IOP was associated with steeper corneal curvature and with younger age and higher blood pressure, or alternatively, with higher estimated CSFP. Corneal curvature radius should be included in the correction of IOP measurements. The potential association between IOP and CSFP as also assumed in adults may warrant further research.

## Introduction

Intraocular pressure (IOP) is one of an important variable for the physiology and pathophysiology of the eye. Its normal distribution and its associations with other ocular and systemic parameters have been examined in numerous preceding investigations [1]–[3]. Few studies, however, were focused on the IOP in children [4]–[18]. These studies had limitations such as a hospital-based study design and inclusion of only a relatively small number of children, and a multivariate analysis was either not performed or did not contain the majority of known factors influencing IOP. In addition, there was no information available for children in China, in particular not from the vast West China. We therefore performed this study on children in western China, measured the IOP and correlated the measurements with ocular and systemic variables. As study region we chose an oasis city in the mid of the Gobi Desert. This oasis city of Ejinaqi had the advantage that due to its isolated location, the exchange of the population with other regions was limited and that the population was relatively constant.

## Methods

The Desert Gobi Children Eye Study was a cross-sectional, school-based study which was performed in city oasis of Ejinaqi, locating in the most western part of the Chinese province of Inner Mongolia. The Ethics Board of the Affiliated Hospital of Inner Mongolia Medical University Hohhot and the local Administration of the Education and School Board of Ejinaqi approved the study and informed written consent was obtained from the parents or guardians of all children. Ejinaqi oasis in the Gobi desert is located in the Ejinaqi region which covers an area of 114,000km^2^ in the western part of the Chinese province of Inner Mongolia. The territory of the oasis stretches from 100.90° to 101.42° East longitude and from 41.85° to 42.50° North latitude. With extremely arid conditions, the study area belongs to the north temperature climate zone with a mean annual precipitation of approximately 40 mm and a mean pan evaporation of 3700 and 4000 mm. Average winter temperature minimums are close to −40°C, while summertime temperatures are warm to hot, with highs that range up to 50°C. The main vegetation are poplar trees. The study included all three schools in Ejinaqi which has a total population of 18,030 inhabitants (including 11,301 Han Chinese, 6209 Mongols and 520 individuals from other minorities). The next settlement is located in a distance of approximately 400 km. Ejinaqi can be reached by train (15 hours from Hohhot, the capital of Inner Mongolia) and by road.

The three schools in Ejinaqi (Ejinaqi primary school (911 students), Ejinaqi middle school (765 students), and Minority school (235 students)) included altogether 1911 children with all children from Ejinaqi attending one of the three schools. All children underwent an ophthalmological examination including measurement of presenting visual acuity and uncorrected visual acuity, a slit lamp-based examination of the anterior ocular segment, and tonometry. IOP was measured by a non-contact tonometer (Canon TX-F Full-Auto Tonometer, Canon Co., Tokyo, Japan). Ocular motility, binocularity and presence of strabismus was examined. After instilling 1% cyclopentolate eye drops (Alcon, Ft. Worth, USA) at least three times, cycloplegia was achieved and auto-refractometry was performed (ARK-900, NIDEK, Tokyo, Japan). Each eye was measured at least 3 times. The spherical equivalent of the refractive error was defined as the spherical value of refractive error plus one half of the cylindrical value. After medical mydriasis, ophthalmoscopy was carried out for examination of the fundus.

The non-ophthalmological examination included measurement of body height (using a stadiometer) and body weight, heart rate and blood pressure (using an automatic blood pressure monitor (YE655A, YUYUE, Jiangsu, China). The body mass index was calculated as the ratio of body weight (expressed in kg) divided by the square of body height (expressed in m).

Using the measurements of diastolic blood pressure and body mass index, we estimated the cerebrospinal fluid pressure (CSFP) using the formula of CSFP [mmHg]  =  0.44 × Body Mass Index [kg/m^2^] + 0.16 × Diastolic Blood Pressure [mmHg] – 0.18 × Age [Years] - 1.91 [19]–[21]. Previous studies had shown that the higher CSFP was associated with higher body mass index, higher diastolic blood pressure and younger age [22].

Statistical analysis was performed using a commercially available statistical software package (SPSS for Windows, version 21.0, IBM-SPSS, Chicago, IL). The normal distribution of data was tested using the Kolmogorov-Smirnov test. As a first step of the statistical analysis, we calculated the mean and standard deviations of the parameters. As a second step, we search for associations between IOP and other ocular and systemic parameters in a univariate analysis. As a first step, we performed a multivariate regression analysis, with IOP as the dependent variable and all those parameters as independent variables which were significantly associated with IOP in the univariate analysis (indicated by a *P*-value ≤0.10). All *P*-values were based on two-sided test and were considered statistically significant if less than 0.05.

## Results

Out of 1911 children who were primarily eligible for the study, 346 refused the examination, so that the study eventually included 1565 (81.9%) children (801 (51.2%) boys) with a mean age of 11.9±3.5 years (median: 11.7 years; range: 6 to 21 years) (Table 1). Mean spherical refractive error was −1.58±2.00 diopters (median: −1.00 diopters; range: −13.75 to +5.50 diopters) in the right eye and −1.54±2.04 diopters (median: −0.75 diopters; range: −25.50 to +6.25 diopters) in the left eye. Seventeen (1.0%) of the children had a myopic refractive error exceeding -8 diopters.

**Table 1:** General information of subjects.

	Number (%)			Systolic Blood Pressure (mm Hg)	Diastolic Blood Pressure (mm Hg)	Intraocular Pressure (Right Eye) (mmHg)	Intraocular Pressure (Left Eye) (mm Hg)	Body Mass Index (kg/m^2^)
Age (Years)	Boys	Girls	Total					
6–10	342	335	677	106.5±10.2	66.1±8.4	17.3±3.5	17.4±3.5	17.0±2.9
11–14	270	265	535	116.1±9.8	70.6±8.2	17.1±3.4	17.1±3.6	20.1±3.9
15–21	189	164	353	120.9±10.1	73.0±8.5	16.6±3.3	16.4±3.5	21.6±4.0
**Total**	801 (51.2)	764 (48.8)	1565 (100.0)	113.0±11.7	69.2±8.8	17.1±3.6	17.1±3.4	19.1±4.0

**Figure 1:**
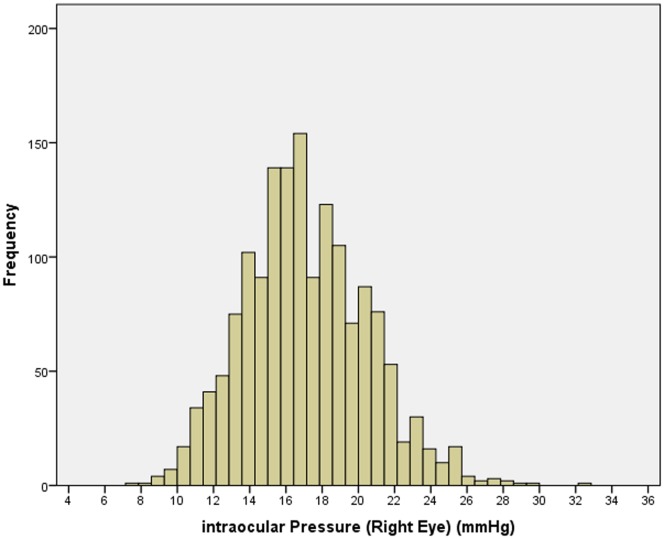
Histogram Showing the Distribution of Intraocular Pressure in the Gobi Desert Children Eye Study.

Mean IOP was 17.1±3.6 mm Hg (median: 16.8 mm Hg; range: 5.6 to 31.5 mm Hg) in the right eye and 17.1±3.4 mm Hg (median: 16.9 mm Hg; range: 7.8 to 32.3 mm Hg) in the left eye. For both eyes, the distribution of IOP showed approximately a Gaussian distribution curve with a minor skew to the right (Fig. 1). For both eyes, IOP was not normally distributed (Kolmogorov-Smirnov test; *P*<0.001). Mean body mass index (BMI) was 19.1±4.0 kg/m^2^, with 6.2% of the children having a BMI higher than 25 and ≤30, and 1.5% of the children having a BMI higher than 30.

When divided into three age groups, mean systolic and diastolic blood pressure and mean body mass index increased with older age group while the mean IOP decreased (Table 1). Within all three age groups, higher IOP was significantly associated with higher blood pressure (all *P*<0.05).

**Figure 2:**
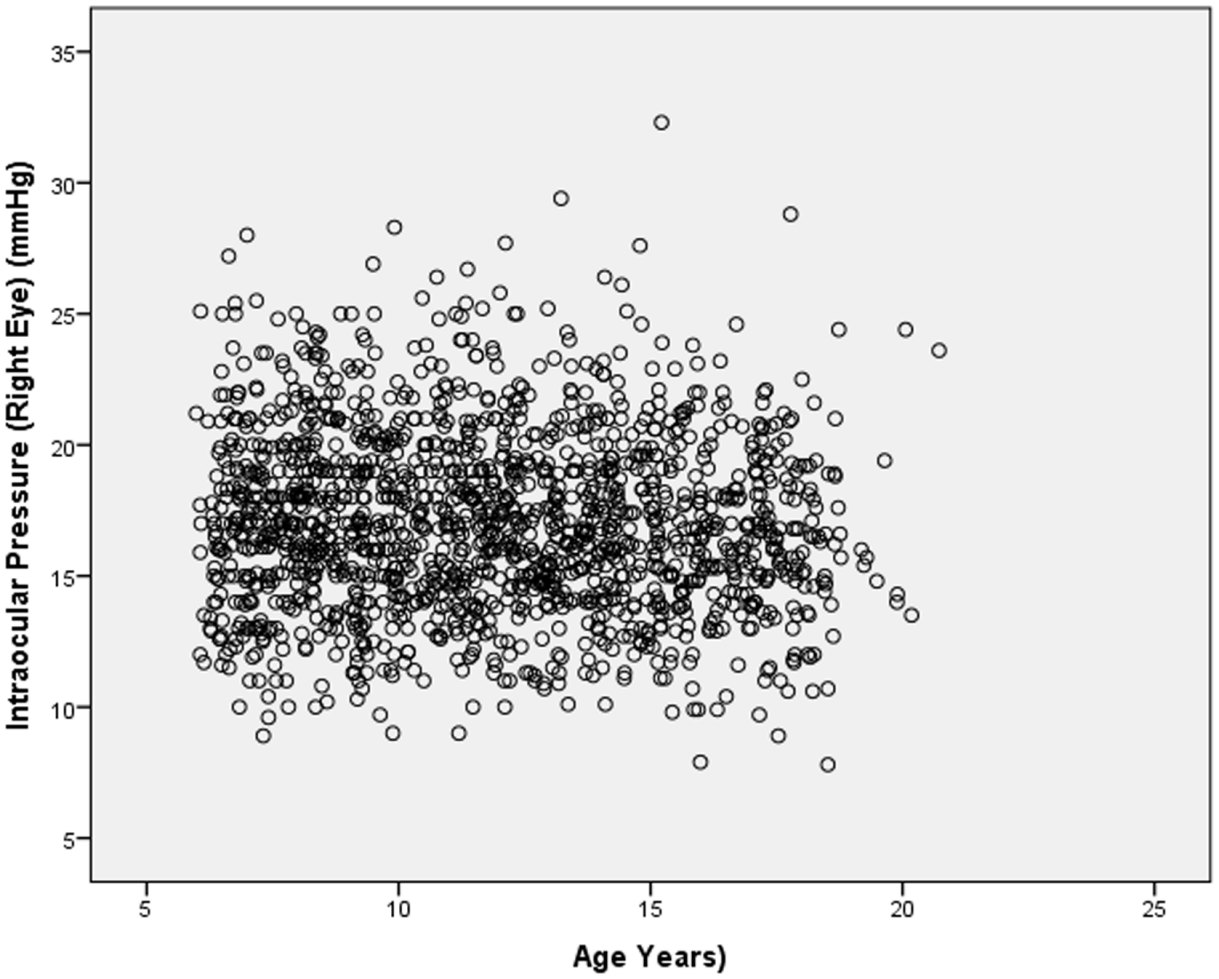
Scattergram Showing the Distribution of Intraocular Pressure and Age in the Gobi Desert Children Eye Study.

**Figure 3:**
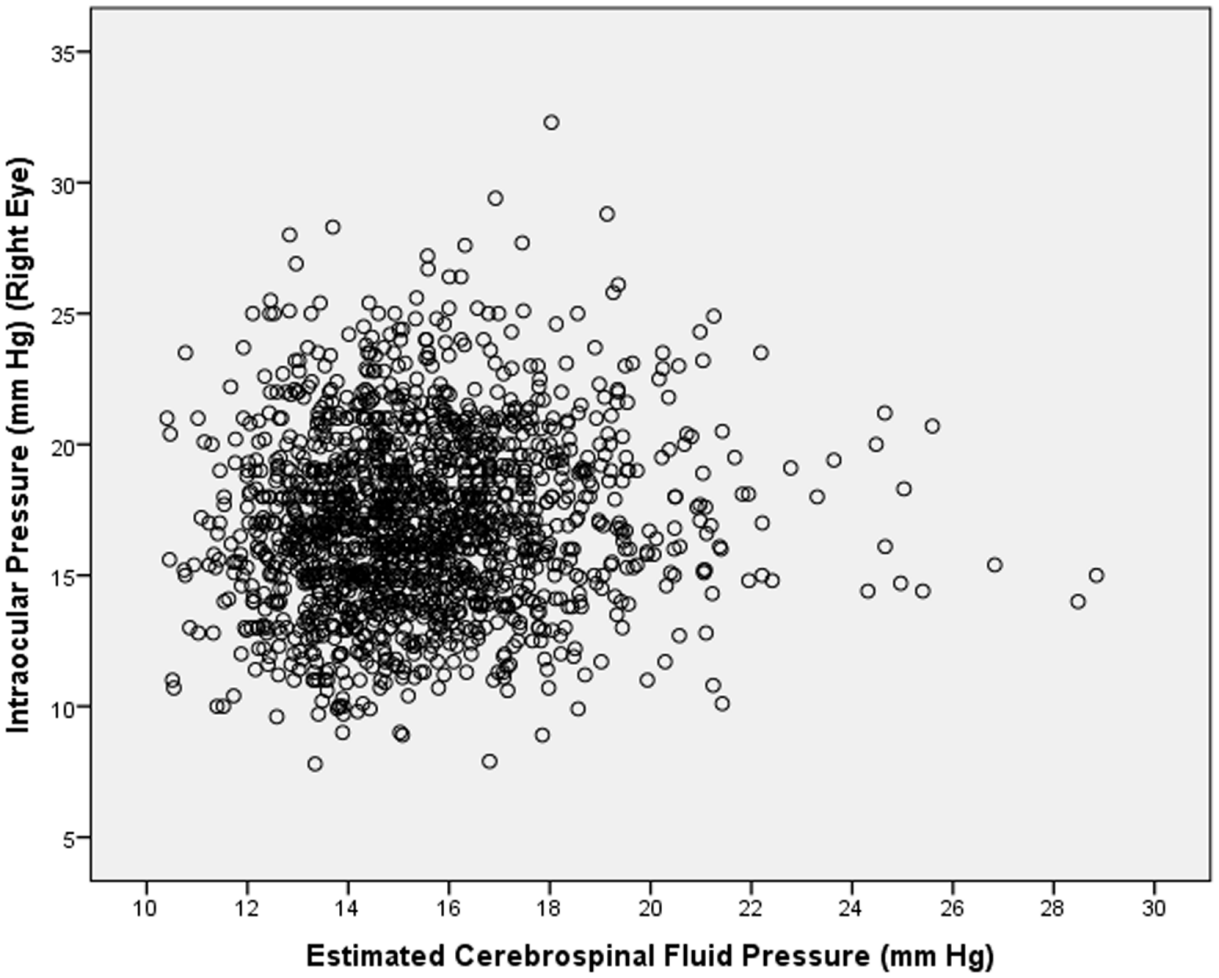
Scattergram Showing the Distribution of Intraocular Pressure and Estimated Cerebrospinal Fluid Pressure Age in the Gobi Desert Children Eye Study.

In univariate linear analysis, IOP decreased significantly with older age (*P*<0.001; standardized coefficient beta: −0.07; regression coefficient B: −0.07; 95%CI: −0.11, −0.02) (Fig. 2). In univariate analysis, higher IOP was significantly associated with female gender (*P* = 0.02), higher blood pressure (*P<*0.001), higher estimated CSFP (*P*<0.001) (Fig. 3), Han Chinese ethnicity of the father (*P* = 0.04), and higher keratometric readings (*P<*0.001) (Table 2).

**Table 2:** Associations (Univariate Analysis) between Intraocular Pressure (Right Eyes) and Systemic and Ocular Parameters in the Gobi Desert Children Eye Study.

Parameter	*P*-Value	Standardized Coefficient Beta	Regression Coefficient B	95% Confidence Interval
Age (Years)	<0.001	−0.07	−0.07	−0.11, −0.02
Gender	0.02	0.06	0.42	0.08, 0.76
Body Height (cm)	0.05	−0.05	−0.01	−0.02, 0.00
Body Weight (kg)	0.60	−0.01	−0.003	−0.013, 0.007
Body Mass Index (kg/m^2^)	0.46	0.02	0.02	−0.03, 0.06
Systolic Blood Pressure (mm Hg)	<0.001	0.10	0.03	0.01, 0.04
Diastolic Blood Pressure (mm Hg)	<0.001	0.10	0.04	0.02, 0.06
Mean Blood Pressure (m Hg)	<0.001	0.11	0.04	0.02, 0.06
Estimated Cerebrospinal Fluid Pressure (mm Hg)	<0.001	0.09	0.13	0.06, 0.20
Fatheŕs Ethnicity (Han Chinese Versus Mongolian)	0.049	0.05	0.43	0.001, 0.86
Motheŕs Ethnicity (Han Chinese Versus Mongolian)	0.16	0.04	0.29	−0.11, 0.70
Gestational Time	0.37	0.02	0.01	−0.01, 0.02
Oxygen Supply in the Neonatal Phase	0.40	−0.02	−0.29	−0.95, 0.38
Number of Hours Spent Indoors (Hours)	0.91	−0.003	−0.01	−0.14, 0.12
Number of Hours Spent Outdoors (Hours)	0.66	0.01	0.03	−0.10, 0.16
				
Keratometric Reading (Diopters)	<0.001	0.12	0.26	0.15, 0.36
Refractive Error (Spherical Equivalent) (Diopters)	0.13	−0.04	−0.07	−0.15, 0.02
Intraocular Pressure Contralateral Eye (mm Hg)	<0.001	0.74	0.71	0.68, 0.74
Best Corrected Visual Acuity	0.78	−0.01	−0.01	−0.05, 0.04

We then performed a multivariate linear regression analysis, which included intraocular pressure as the dependent variable and all those parameters as independent variables which were associated with intraocular pressure in the univariate analysis with a *P*-value ≤0.10. In a first step, we dropped the parameters of mean blood pressure, systolic blood pressure and body weight from the list of independent variables due to the collinearity with diastolic blood pressure and with body mass index. We then dropped in a combination of stepwise, forward and backward regression analysis, all those parameters which were no longer significantly associated with IOP in the multivariate analysis. In the final model, higher IOP of the right eye was significantly associated with the non-ocular parameters of younger age (*P*<0.001), higher diastolic blood pressure (*P*<0.001), higher corneal refractive power (*P*<0.001), more myopic refractive error (*P* = 0.035), and Han Chinese ethnicity of the father (*P* = 0.03) (Table 3). If age and diastolic blood pressure were dropped and replaced by the estimated CSFP, higher IOP was associated with higher estimated CSFP (*P*<0.001; beta: 0.09; B: 0.13; 95%CI: 0.06, 0.21), higher corneal refractive power (*P*<0.001; beta: 0.12; B: 0.26; 95%CI: 0.15, 0.37), and Han Chinese ethnicity of the father (*P* = 0.04; beta: 0.05; B: 0.44; 95%CI: 0.02, 0.87).

**Table 3:** Associations (Multivariate Analysis) between Intraocular Pressure (Right Eyes) and Systemic and Ocular Parameters in the Gobi Desert Children Eye Study.

Parameter	*P*-Value	Standardized Coefficient Beta	Regression Coefficient B	95% Confidence Interval
Age (Years)	<0.001	−0.13	−0.13	−0.18, −0.07
Diastolic Blood Pressure (mm Hg)	<0.001	0.13	0.05	0.03, 0.07
Corneal Refractive Power (Diopters)	<0.001	0.11	0.23	0.12, 0.34
Refractive Error (Spher. Equiv.) (Diopters)	0.035	−0.06	−0.10	−0.19, −0.001
Fatheŕs Ethnicity (Han Chinese/Non-Han Chinese)	0.03	0.06	0.42	0.04, 0.89

If the IOP of the left eye was taken as dependent variable in the multivariate analysis, similar results were obtained: higher IOP of the left eyes was associated with younger age (*P*<0.001), higher diastolic blood pressure (*P*<0.001), higher corneal refractive power (*P*<0.001), and additionally, with female gender (*P*<0.001) (Table 4). If diastolic blood pressure was dropped and replaced by estimated CSFP, higher IOP was associated with younger age (*P*<0.001; beta: −0.13; B: −0.13; 95%CI: −0.18, −0.08), female gender (*P*<0.001; beta: 0.10; B: 0.70; 95%CI: 0.34, 1.06), higher corneal refractive power (*P*<0.001; beta: 0.08; B: 0.21; 95%CI: 0.08, 0.34), and higher estimated CSFP (*P*<0.001; beta: 0.12; B: 0.18; 95%CI: 0.10, 0.25).

**Table 4:** Associations (Multivariate Analysis) between Intraocular Pressure (Left Eyes) and Systemic and Ocular Parameters in the Gobi Desert Children Eye Study.

Parameter	*P*-Value	Standardized Coefficient Beta	Regression Coefficient B	95% Confidence Interval
Age (Years)	<0.001	−0.14	−0.13	−0.19, −0.08
Gender (Boys/Girls)	<0.001	0.09	0.67	0.32,1.03
Diastolic Blood Pressure (mm Hg)	<0.001	0.13	0.05	0.03, 0.07
Corneal Refractive Power (Diopters)	0.002	0.08	0.21	0.08, 0.33

## Discussion

In our population-based study on school children in an oasis in the Gobi Desert, higher IOP was significantly associated with younger age, higher diastolic blood pressure, steeper cornea and more myopic refractive error. If diastolic blood pressure were dropped from the analysis in the otherwise unchanged statistical model, higher IOP was significantly (*P*<0.001) associated with higher estimated CSFP. In the multivariate model, IOP was not significantly associated with BMI.

The association between higher IOP and higher blood pressure as found in our children study was in agreement with previous population based studies on adults, such as the Rotterdam Study, the Singaporean Tanjong Pagar Study, the Blue Mountains Eye Study, the Beaver Dam Eye Study, and the Los Angeles Latino Eye Study [19]–[24]. In univariate analysis, IOP increased by 0.4 mm Hg for each increase in diastolic blood pressure by 10 mm Hg (Table 2). In the multivariate model, IOP increased by 0.5 mm Hg for ach increase in diastolic blood pressure by 10 mm Hg (Table 4). It confirms the results of the preceding studies and extends their findings onto children. It shows that independently of age, IOP and blood pressure are connected to each other. It is in agreement with the experimental study by Samuels and colleagues who stereotaxically microinjected the gamma-aminobutyric acid receptor antagonist bicuculline methiodide into the dorsomedial and perifornical hypothalamus of rats and a significant increases in heart rate, mean arterial blood pressure, IOP and intracranial pressure [29].

The association between IOP and steeper cornea (i.e., higher corneal refractive power) again agrees with preceding studies on adults in which similar correlation have been reported [28], [30]. The higher the corneal refractive power was, i.e., the steeper the cornea was, the higher were the intraocular pressure readings. The finding of our study may be due to geometrical reasons, since a flat structure as compared with a steep structure needs less external pressure to be further flattened up to a standardized applanation area. The clinical importance of the finding is that IOP measurements should be corrected for central corneal thickness and corneal curvature, in after corneal refractive surgery for the correction of myopia.

In contrast to previous studies on adults, IOP was not significantly associated with BMI in our study population [31]–[34]. In the previous studies, higher body mass index was associated with higher blood pressure, higher IOP and higher CSFP, which in turn was correlated with IOP and blood pressure [33], [34]. The reason for the discrepancy between the previous studies on adults and our study on children may that most of the children in our study had a normal body mass index, with only approximately 8% of the children having overweight or being obese. Also in contrast to previous studies on adults [15], [28], myopic refractive error was only weakly associated with higher IOP in the multivariate analysis in our study. In our study population, only 1.0% of the children had a myopic refractive error exceeding -8 diopters, and only 2.9% of the children had a myopic refractive error exceeding -6 diopters. These figures are markedly lower than those obtained from school children in metropolitan regions at the East coast of China. The relatively low prevalence of high myopia in our study population may have prevented a statistically stronger influence on IOP in the multivariate models. Interestingly, as in our study, the Los Angeles Latino Eye Study neither found a strong association between IOP and myopia [26].

IOP decreased with older age in our study population (Tables 2–4). These results agree with findings of some studies, and are contradictory to results other investigations. An increase in IOP with older age for 405 children up to an age of 12 years was reported by Sihota and colleagues [13]. An increase in IOP with older age for children aged less than 10 years was also reported by Duckman and colleagues [5]. In a similar manner, studies reported on different association between higher age and IOP in adults, with increased IOP in Westerners and decreasing IOP with older age in Japanese [26], [35].

Higher IOP was associated with higher estimated CSFP in the multivariate analysis in our study, if diastolic blood pressure or additionally age (which were included in the formula to calculate the CSFP). With all limitations of the formula to estimate the CSFP, the finding may point to an association between IOP and CSFP. A similar relationship has been measured in adults using lumbar pressure measurements of CSFP, and has also been postulated in other population-based studies of various ethnicities [20]–[22].

Potential limitations of our study should be mentioned. First, the oasis city of Ejinaqi in West China is not representative for whole China. For the vast Western region of China, however, our study was the first one to report on IOP in children. Second, as in any population-based study, the participation rate is of concern. In our study, the response rate of 81.9% of eligible children participating may be sufficient as basis for a population-based statistical analysis. Third, we did not measure central corneal thickness which without doubt influences the IOP measurements. Previous studies on adults showed however, that with or without inclusion of central corneal thickness into the multivariate model, higher IOP was associated with higher blood pressure and flatter cornea shape [23]–[28], [30]. Fourth, we did not asses the educational level of the parents which could also have influenced the IOP. Strengths of our study were that we included almost all children of the region in contrast to previous school-based studies, in which usually schools were randomly selected and their children were asked to participate in the study.

In conclusion, higher IOP in school children in West China was associated with steeper corneal curvature and with younger age and higher blood pressure, or alternatively, with higher estimated CSFP. Corneal curvature radius should be included for the correction of IOP measurements. The potential association between IOP and CSFP as also assumed in adults may warrant further research.
